# Detection of obstructive sleep apnea using Belun Sleep Platform wearable with neural network-based algorithm and its combined use with STOP-Bang questionnaire

**DOI:** 10.1371/journal.pone.0258040

**Published:** 2021-10-11

**Authors:** Eric Yeh, Eileen Wong, Chih-Wei Tsai, Wenbo Gu, Pai-Lien Chen, Lydia Leung, I-Chen Wu, Kingman P. Strohl, Rodney J. Folz, Wail Yar, Ambrose A. Chiang

**Affiliations:** 1 Division of Pulmonary, Critical Care, and Sleep Medicine, University Hospitals Cleveland Medical Center and Department of Medicine, Case Western Reserve University, Cleveland, Ohio, United States of America; 2 Belun Technology Company Limited, Sha Tin, Hong Kong; 3 Department of Computer Science, National Yang Ming Chiao Tung University, Hsinchu, Taiwan; 4 FHI360, Durham, NC, United States of America; 5 Division of Sleep Medicine, Louis Stokes Cleveland VA Medical Center, Cleveland, Ohio, United States of America; 6 Department of Family Medicine, University Hospitals Cleveland Medical Center, Cleveland, Ohio United States of America; Brigham and Women’s Hospital and Harvard Medical School, UNITED STATES

## Abstract

Many wearables allow physiological data acquisition in sleep and enable clinicians to assess sleep outside of sleep labs. Belun Sleep Platform (BSP) is a novel neural network-based home sleep apnea testing system utilizing a wearable ring device to detect obstructive sleep apnea (OSA). The objective of the study is to assess the performance of BSP for the evaluation of OSA. Subjects who take heart rate-affecting medications and those with non-arrhythmic comorbidities were included in this cohort. Polysomnography (PSG) studies were performed simultaneously with the Belun Ring in individuals who were referred to the sleep lab for an overnight sleep study. The sleep studies were manually scored using the American Academy of Sleep Medicine Scoring Manual (version 2.4) with 4% desaturation hypopnea criteria. A total of 78 subjects were recruited. Of these, 45% had AHI < 5; 18% had AHI 5–15; 19% had AHI 15–30; 18% had AHI ≥ 30. The Belun apnea-hypopnea index (bAHI) correlated well with the PSG-AHI (*r* = 0.888, *P* < 0.001). The Belun total sleep time (bTST) and PSG-TST had a high correlation coefficient (*r* = 0.967, *P* < 0.001). The accuracy, sensitivity, specificity in categorizing AHI ≥ 15 were 0.808 [95% CI, 0.703–0.888], 0.931 [95% CI, 0.772–0.992], and 0.735 [95% CI, 0.589–0.850], respectively. The use of beta-blocker/calcium-receptor antagonist and the presence of comorbidities did not negatively affect the sensitivity and specificity of BSP in predicting OSA. A diagnostic algorithm combining STOP-Bang cutoff of 5 and bAHI cutoff of 15 events/h demonstrated an accuracy, sensitivity, specificity of 0.938 [95% CI, 0.828–0.987], 0.944 [95% CI, 0.727–0.999], and 0.933 [95% CI, 0.779–0.992], respectively, for the diagnosis of moderate to severe OSA. BSP is a promising testing tool for OSA assessment and can potentially be incorporated into clinical practices for the identification of OSA.

**Trial registration:** ClinicalTrial.org NCT03997916 https://clinicaltrials.gov/ct2/show/NCT03997916?term=belun+ring&draw=2&rank=1

## Introduction

Obstructive Sleep Apnea (OSA) is a challenging sleep disorder associated with increased cardiovascular and metabolic morbidities as well as increased mortality [1–7]. In the general adult population, the prevalence of OSA defined by apnea-hypopnea index (AHI) ≥ 5 events/h ranges from 9% to 38% and the prevalence is likely to further increase due to both the obesity epidemic and aging of society [8–10].

The current gold standard for the diagnosis of OSA requires overnight multi-channel polysomnography (PSG) in the sleep lab. Type 3 home sleep apnea testing (HSAT) devices are now widely used despite concerns of false negative results [11–14]. Technologies that analyze peripheral arterial tone (PAT) signal are considered acceptable alternatives [15, 16]. In the recent Peripheal Arterial Tonometry Evaluation of Reliability (PATER) study, Ioachimescu et al. conducted a large cohort comparing WatchPAT with synchronous PSG in 500 consecutive veterans [17]. Using a 3% desaturation threshold, the WatchPAT-200 automated algorithm overestimated severity by an average of +4 events/h, and the 4% threshold underestimated severity by −6 events/h. Diagnostic concordance was found in 42%, 41%, and 83% of mild, moderate, and severe OSA. These authors recommended that those with no OSA or mild OSA, assessed by WatchPAT, undergo in-lab PSG testing.

**Fig 1:**
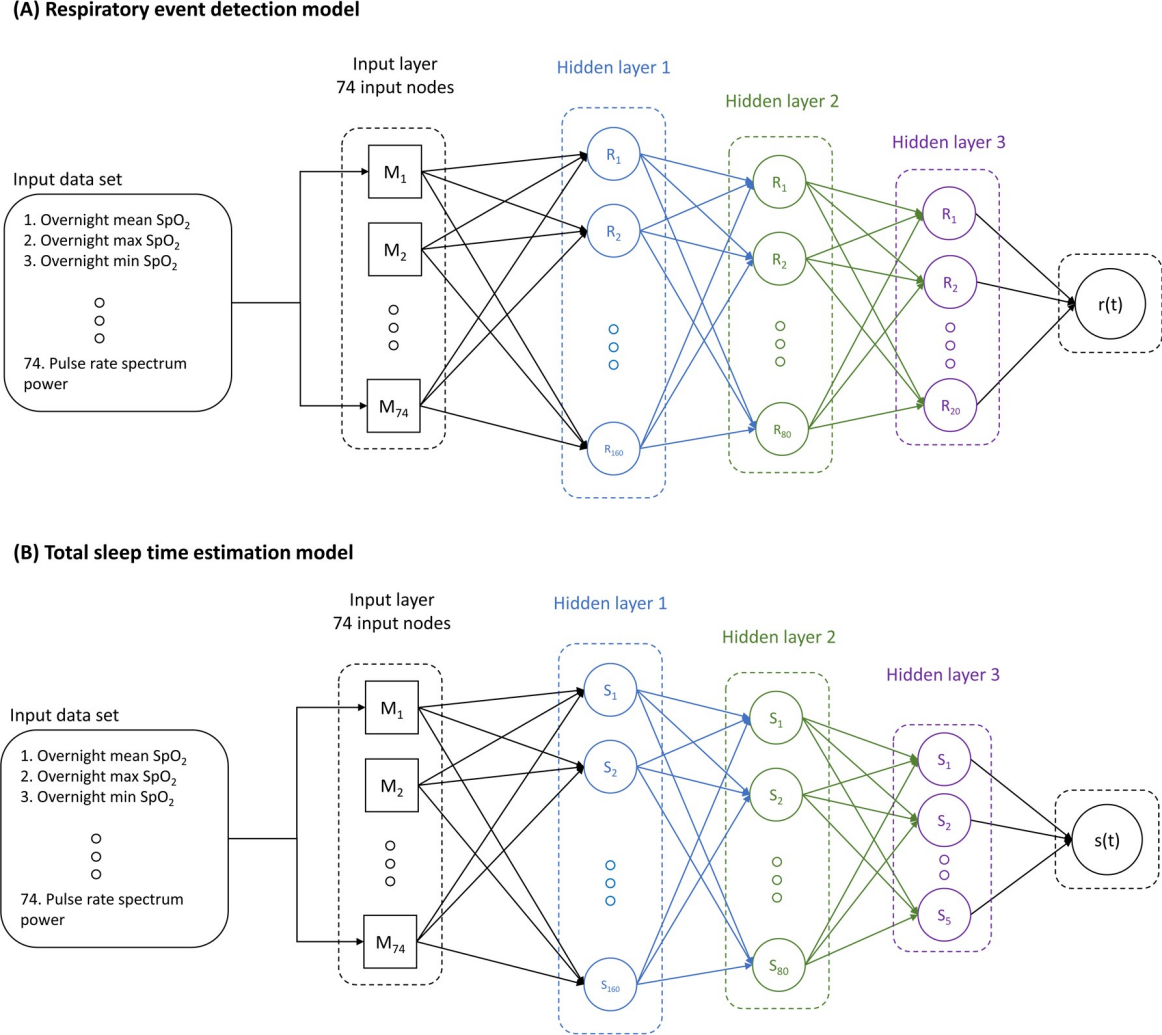
BSP neural network architecture diagram. The BSP proprietary OSA detection algorithm was built using neural networks and trained with a dataset of 5,783 patients and 8,417 records of overnight sleep studies. (A) Respiratory event detection model containing 160, 80, and 20 neurons; (B) Total sleep time estimation model containing 160, 80, and 5 neurons.

Wearable devices now exert a significant impact in medicine and healthcare [18, 19]. Many wearables allow physiological data acquisition in sleep and enable clinicians to assess sleep outside of sleep labs [20–28]. The recent advances of wearable technology arise largely from the miniaturization of biosensors, low-power computation, and the application of artificial intelligence, particularly machine learning [29, 30]. The Belun Sleep Platform (BSP, Belun Technology Company Limited, Hong Kong) consists of a patented wearable ring device, a charging cradle, and cloud-based software. The Belun Ring, an FDA-cleared pulse oximeter, acquires pulse oximetry, photoplethysmography (PPG), and 3-axis accelerometer signals from the radialis indicis artery of the proximal index finger. The BSP proprietary OSA detection algorithm was built using neural networks and trained with a dataset of 5,783 patients and 8,417 records of overnight sleep studies scored with the 4% oxygen desaturation hypopnea criteria [31]. Two fully connected neural networks for respiratory event detection and total sleep time estimation were trained respectively to learn features and patterns from SpO2, pulse rate, heart rate variability (HRV), accelerometry signal, and PPG waveform to detect respiratory events and total sleep time by 5-minute segments. Features were extracted from overnight data, current segment, and consecutive prior segments. Both the respiratory event detection model and total sleep time estimation model have three fully connected hidden layers. The respiratory event detection model contains 160, 80, and 20 neurons in each layer while the total sleep time estimation model contains 160, 80, and 5 neurons (Fig 1). The activation functions used in both models are the sigmoid function. The outputs of the respiratory event detection model are the predicted number of respiratory events in each segment.

A recent proof-of-concept study in adult subjects not taking heart-rate affecting medications and having no significant comorbidities showed a good correlation between the Belun apnea-hypopnea index (bAHI) and the PSG-AHI as well as good sensitivity, specificity, positive predictive value (PPV), and negative predictive value (NPV) in categorizing AHI ≥ 15 events/h. The Belun total sleep time (bTST) also correlated well with the PSG-TST [32].

In this current study, we hypothesized that BSP can reliably identify moderate to severe OSA in a sleep lab patient population including those who take heart rate-affecting medications and have non-arrhythmic comorbidities. We specifically studied the accuracy of bAHI and bTST by comparing them directly to concurrent in-lab PSG-AHI and PSG-TST. We also investigated the performance of a diagnostic algorithm incorporating STOP-Bang to predict moderate to severe OSA. STOP-Bang was selected in this study over Berlin Questionnaire (BQ) and Epworth Sleepiness Score (ESS) as studies have shown that STOP-Bang is more accurate than ESS and BQ in predicting OSA of various severity as ESS only assesses sleepiness and BQ focuses primarily on OSA-related symptoms as opposed to STOP-Bang questionnaire, which includes demographic and anthropometric characteristics such as BMI, age, neck circumference, and gender [33–35]. We hypothesized that the addition of STOP-Bang may improve the diagnostic precision as well as the categorization of OSA severity.

## Methods

### Participants

**Fig 2:**
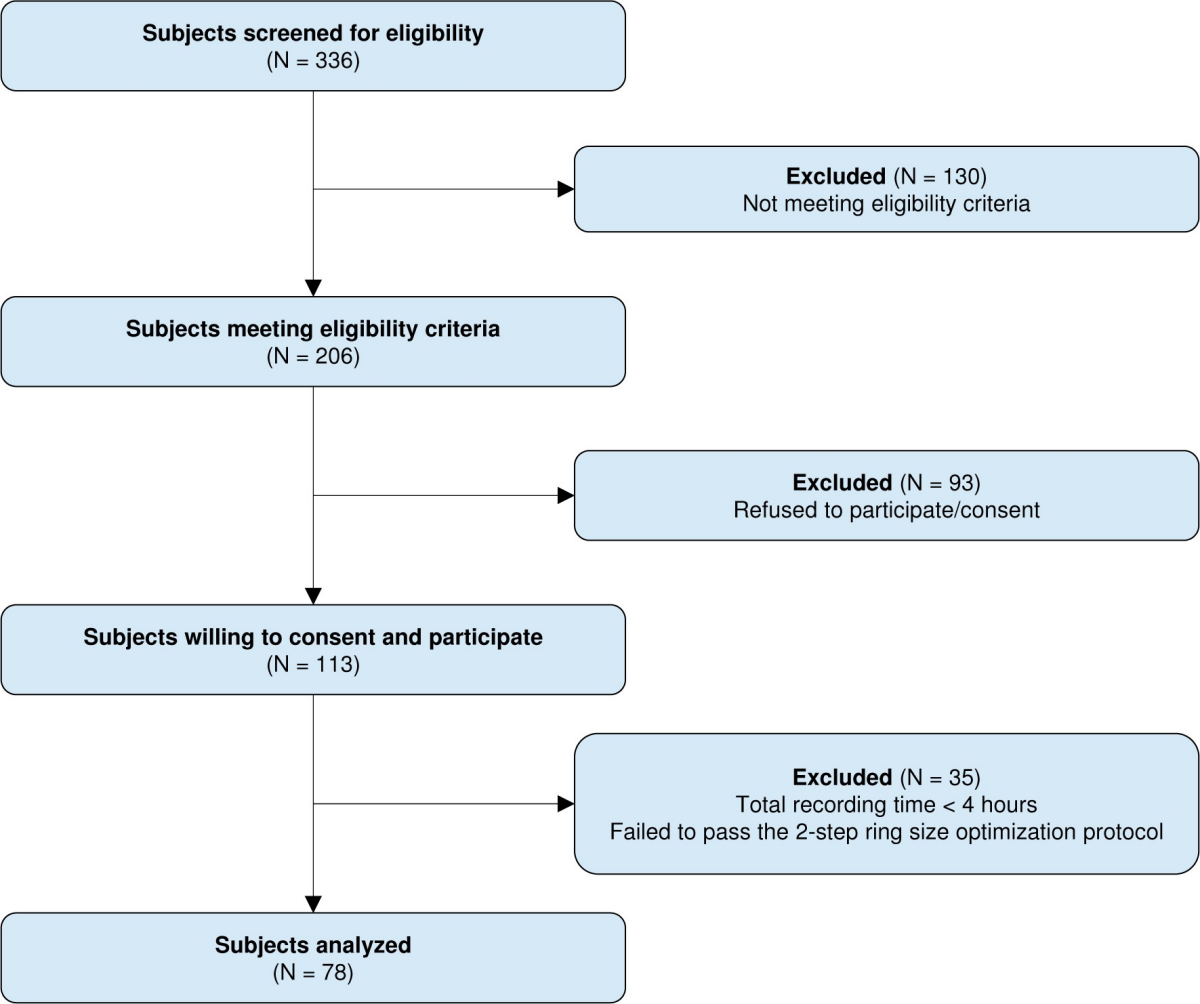
CONSORT flow diagram.

A total of 336 consecutive subjects who were referred to the American Academy of Sleep Medicine (AASM)-accredited University Hospitals Cleveland Medical Center labs (Beachwood and Cleveland, Ohio) for evaluation of sleep disorders were screened; 206 adult subjects met the eligibility criteria; 113 signed informed consent and participated in the study. Seventy-eight subjects who passed the 2-step ring size optimization protocol and had a recording time ≥ 4 hours were enrolled (Fig 2) [32]. Patients who were on heart-rate altering medications such as beta-blockers and/or calcium channel antagonists were included in this study. All underwent BSP testing with simultaneous overnight PSG.

The inclusion criteria were adults age 18–80 with a valid email address who are willing to provide written informed consent and complete STOP-Bang questionnaire. The exclusion criteria included subjects who were referred for positive airway pressure titration study; those with atrial fibrillation, pacemaker, defibrillator, left ventricular assist device, or status post-cardiac transplantation; patients on home O2, non-invasive ventilator, diaphragmatic pacing, or any form of nerve stimulator; patients with hospitalization in the prior 30 days or unstable cardiopulmonary status judged to be unsafe for a sleep study by the sleep tech or the on-call sleep physician on the night of the study; those with heart rate outside the 50–100 per minute range at the time of testing, and those unable to complete the required study. Patients with atrial fibrillation were excluded from this study as the BSP analysis algorithm has not been tested in this specific population.

### BSP algorithm and data collection

In this study, the neural network algorithm used in the BSP is the same as that used in the proof-of-concept study [32]. The BSP ring captures oxygen saturation (SpO2), photoplethysmography (PPG), and accelerometer signals. The bTST is derived from features extracted from accelerometer, SpO2, and PPG signals. The bAHI is derived from bTST and features extracted from heart rate variability and SpO2 changes [32].

STOP-Bang data were collected on the night of the sleep study. There are seven different ring sizes (sizes 5, 7, 9, 10, 11, 13, and 15) available. A 2-step ring selection approach was used to ensure the appropriate ring size as described in the proof-of-concept study [32]. Once the ring selection is optimized, the ring is securely placed on the proximal phalanx of the non-dominant hand index finger and left in place overnight. At the conclusion of the sleep study, the ring is removed and placed back on the cradle connected to a PC laptop using a USB cable. The acquired data are then uploaded to the cloud via the internet and the neural network algorithm automatically calculates and generates a report that can be downloaded immediately. To assess the accuracy of bTST, sleep stages in 30-second epochs from PSG were extracted according to the valid sleep period of BSP for comparison with the PSG-TST. The bAHI is defined as the number of respiratory events estimated by the BSP algorithm divided by the bTST. The PSG scoring technicians and the interpreting physician (AAC) were blinded to the BSP results.

### Polysomnography scoring and statistical analysis

Attended in-lab PSG (SleepWorks, Natus, Pleasanton, CA) was performed in all patients in a standard fashion. The PSG montage includes EEG leads (O1M2, O2M1, C1M2, C2M1, F1M2, F2M1), right electrooculogram, left electrooculogram, chin electromyogram, nasal pressure airflow, thermistor airflow, chest and abdominal respiratory efforts, pulse oximetry, left leg electromyogram, right leg electromyogram, and electrocardiogram. CPAP flow was also monitored for split night studies. All studies were manually scored in 30-sec epochs according to the AASM Scoring Manual, version 2.4 [36, 37]. An obstructive apnea event is defined as a decrease in the thermistor airflow to < 10% of the baseline for ≥ 10 seconds with continued respiratory effort. A hypopnea event is defined as a decrease in nasal pressure signal excursions by 30% to 90% of the baseline for ≥ 10 seconds accompanied by oxygen desaturation ≥ 4%. The Belun Technology Company staff were blinded to the in-lab PSG results.

Statistical analysis was performed using R (R Core Team) to assess the accuracy of BSP in predicting OSA [38]. We used the Kruskal-Wallis test by ranks to compare the medians of age, body mass index (BMI), AHI, central apnea index (CAI), and STOP-Bang score among OSA severities as these data are not normally distributed. Mantel-Haenszel Chi-Squared test was used to compare frequencies in gender, and race among OSA severities [39]. Agreements between results derived from PSG-AHI versus bAHI and PSG-TST versus bTST were determined by Pearson’s correlation and Bland-Altman method [40]. Statistical measures including accuracy, sensitivity, specificity, PPV, NPV, positive likelihood ratio (LR+), negative likelihood ratio (LR-), Cohen’s Kappa coefficient (Kappa), and area under the receiver operator Curve (AUCROC) were computed at PSG-AHI cutoffs of 5, 15, and 30 events/h. Fisher’s exact test was used to compare sensitivity and specificity between subjects with and without heart rate-affecting medications and those with and without comorbidities. Statistical measures of diagnostic algorithms combining STOP-Bang score cutoffs of 3, 4, and 5 with a bAHI cutoff of 15 events/h were compared for accuracy in predicting moderate to severe OSA. The diagnostic algorithm yielding the best results was internally validated using bootstrapping with 1,000 samples [41].

The study protocol was approved by the University Hospitals Institutional Review Board (STUDY20181042). All ongoing and related BSP trials are registered at ClinicalTrials.org. This trial was registered after recruitment of participants began due to clerical error.

## Results

### Baseline characteristics

A total of 78 adult subjects were included. Baseline data including gender, race, BMI, STOP-Bang, AHI, CAI are shown in Table 1. This sampled population was unbalanced and skewed towards female subjects (65%) and those with normal and mild OSA (63%). There were 22% with STOP-Bang score < 3 and only 36% of subjects had STOP-Bang ≥ 5. Concerning comorbidities, 29% of subjects had diabetes, 58% hypertension, 49% hyperlipidemia, and 31% asthma/chronic obstructive pulmonary disease. Echocardiography report was available in 40% of subjects with 4% having systolic dysfunction and 20% having diastolic dysfunction. There were 32% taking beta-blockers and/or calcium channel antagonists.

**Table 1:** Summary of subject characteristics and PSG results.

Parameter		OSA Severity					*P*-value
	ALL	No OSA	Mild OSA	Moderate OSA	Severe OSA	All OSA	
**Subject (%)**	78 (100%)	35 (45%)	14 (18%)	15 (19%)	14 (18%)	43 (55%)	-
**Gender (%)**							< 0.05^b^
**Male**	27 (35%)	9 (12%)	3 (4%)	10 (13%)	5 (6%)	18 (23%)	
**Female**	51 (65%)	26 (33%)	11 (14%)	5 (6%)	9 (12%)	25 (32%)	
**Age (y)**	51.5 ± 13.4	49 ± 14	47 ± 12	54 ± 11	54 ± 15	52 ± 13	0.24^a^
**Race (%)**							0.57^b^
**Black**	41 (53%)	17 (22%)	10 (13%)	6 (8%)	8 (10%)	24 (31%)	
**White**	4 (5%)	3 (4%)	0 (0%)	1 (1%)	0 (0%)	1 (1%)	
**Asian**	30 (38%)	13 (17%)	3 (4%)	8 (10%)	6 (8%)	17 (22%)	
**Others**	3 (4%)	2 (2%)	1 (1%)	0 (0%)	0 (0%)	1 (1%)	
**BMI (kg/m** ^**2**^ **)**	36.1 ± 10.1	31.4 ± 7.9	42.5 ± 10.2	39.1 ± 10.6	38.1 ± 9.8	39.9 ± 10.1	<0.01^a^
**STOP-Bang**	3.9 ± 1.8	3.1 ± 1.6	3.8 ± 1.5	5.3 ± 1.5	4.4 ± 1.9	4.5 ± 1.7	<0.01^a^
**AHI (/h)**	14.5 ± 17.0	2.4 ± 1.5	8.3 ± 2.0	21.1 ± 3.9	43.6 ± 16.9	24.9 ± 20.0	<0.001^a^
**CAI (/h)**	1.39 ± 3.5	0.40 ± 0.7	0.77 ± 1.8	2.11 ± 3.9	3.71 ± 6.6	2.19 ± 4.6	<0.05^a^

AHI = apnea-hypopnea index; BMI = body mass index; CAI = central apnea index; OSA = obstructive sleep apnea; PSG = polysomnography. No OSA = AHI < 5; Mild OSA = AHI 5 to < 15; Moderate OSA = AHI 15 to < 30; Severe OSA = AHI ≥ 30.

Mean ± standard deviation for age, BMI, AHI, and STOP-Bang.

Numbers of subjects (%) for gender and race.

^a^ Kruskal-Wallis test by ranks to compare medians of age, BMI, STOP-Bang, AHI, and CAI among No OSA, Mild OSA, Moderate OSA, and Severe OSA groups.

^b^ Mantel-Haenszel Chi-Squared test to compare frequencies in gender, and race among No OSA, Mild OSA, Moderate OSA, and Severe OSA groups.

### Comparing BSP to PSG

**Fig 3:**
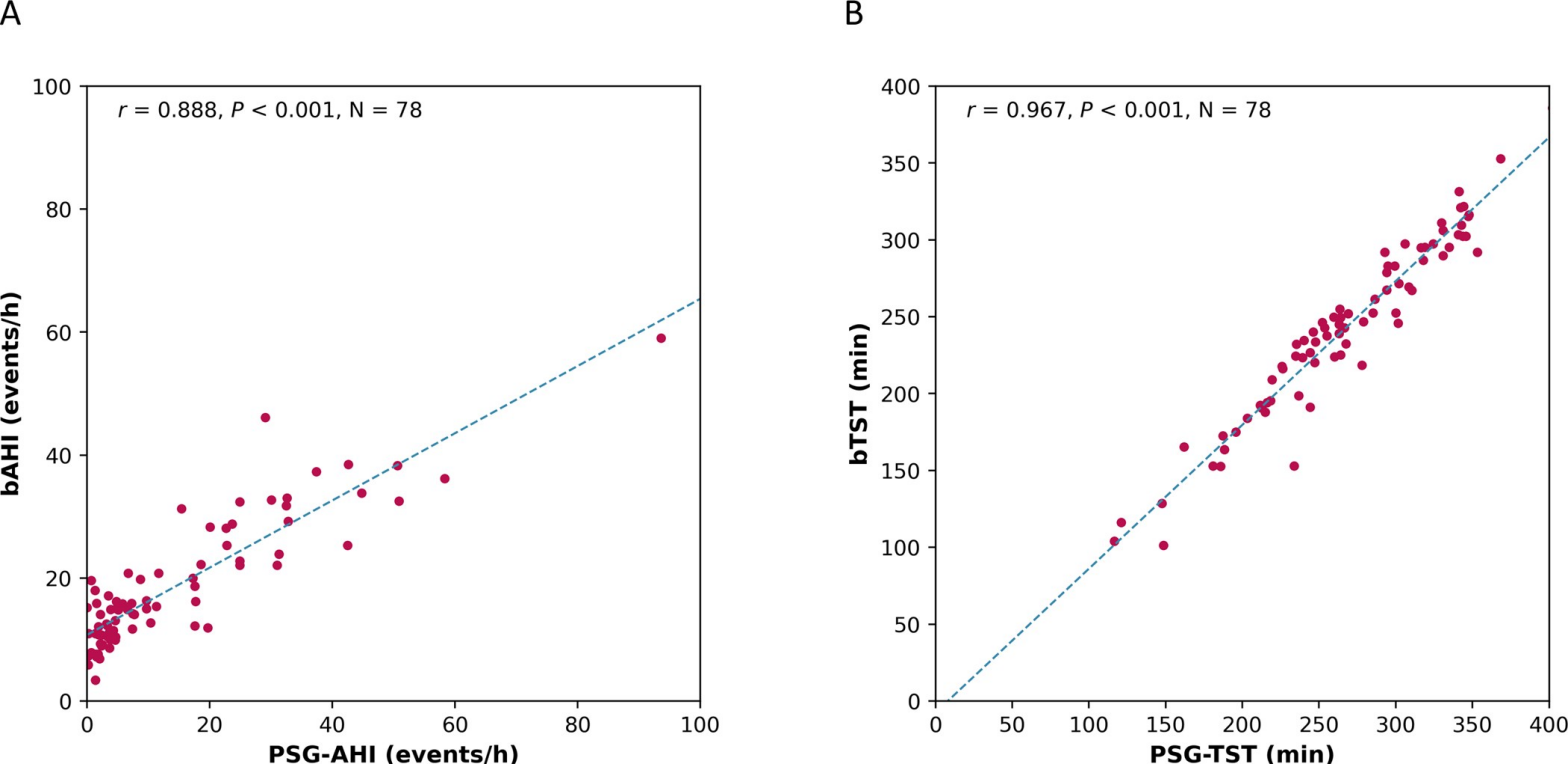
Scatterplots. (A) Scatterplot comparing bAHI to PSG-AHI; (B) Scatterplot comparing bTST to PSG-TST. bAHI = Belun apnea-hypopnea index; PSG-AHI = polysomnography apnea-hypopnea index; bTST = Belun total sleep time; PSG-TST = polysomnography total sleep time.

**Fig 4:**
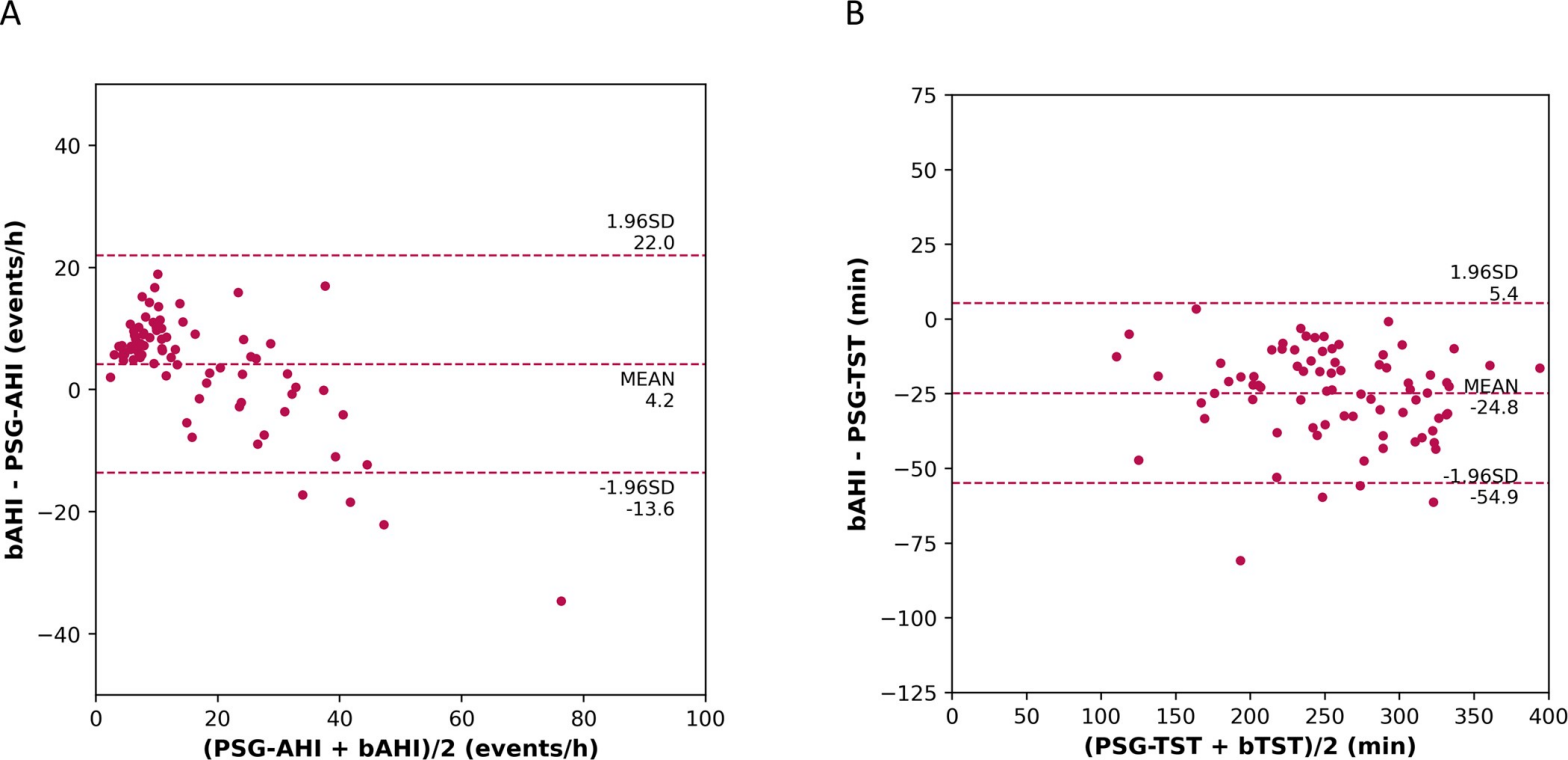
Bland-Altman plots. (A) Bland-Altman plot for bAHI vs. PSG-AHI; (B) Bland-Altman plot for bTST vs. PSG-TST. bAHI = Belun apnea-hypopnea index; PSG-AHI = polysomnography apnea-hypopnea index; bTST = Belun total sleep time; PSG-TST = polysomnography total sleep time.

**Fig 5:**
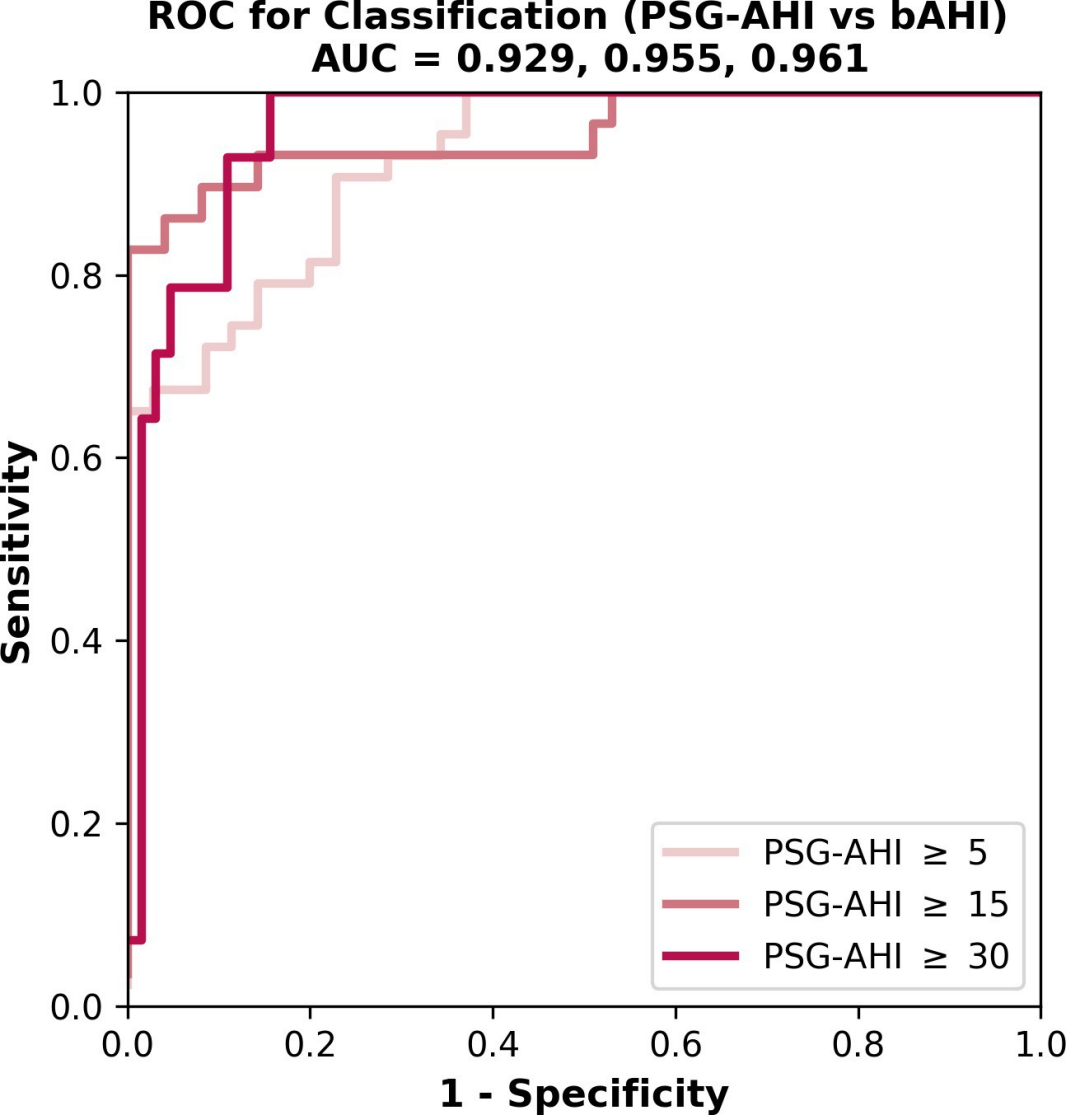
ROC curves for bAHI vs. PSG-AHI at PSG-AHI Cutoffs of 5, 15, and 30 events/h (N = 78). ROC = receiver operator characteristic; bAHI = Belun apnea-hypopnea index; PSG-AHI = polysomnography apnea-hypopnea index.

The bAHI correlated well with the PSG-AHI (*r* = 0.888, *P* < 0.001; Fig 3A). The mean difference between bAHI and PSG-AHI was +4.2 events/h with a 1.96 standard deviation of 17.8 events/h (Fig 4A). The bTST is also highly correlated with PSG-TST (*r* = 0.967, *P* < 0.001; Fig 3B). The mean difference between bTST and PSG-TST was -24.8 minutes with a 1.96 SD of 30.2 minutes (Fig 4B). The receiver operator curve (ROC) was plotted at three different PSG-AHI cutoffs of 5, 15, and 30 events/h and is shown in Fig 5. The overall performance of the BSP including accuracy, sensitivity, specificity, PPV, NPV, LR+, LR-, kappa, and AUROC for AHI of 5, 15, and 30 events/h are summarized in Table 2. The optimal bAHI cutoff for prediction of moderate or severe OSA was 16 events/h with a sensitivity, specificity, PPV, and NPV of 0.929, 0.820, 0.744, and 0.954. The contingency table comparing OSA severity measured by PSG and BSP is shown in Table 3. Statistical measures for the subjects taking heart-rate affecting medications and for those with comorbidities are summarized in Table 4. Fisher’s exact test indicated no significant difference in sensitivity and specificity between subjects with and without heart rate-affecting medications (*P* = 0.192 for sensitivity; *P* = 0.474 for specificity). Comorbidities also did not affect the performance of BSP testing (*P* = 1.000 for sensitivity; *P* = 0.410 for specificity).

**Table 2:** Performance (mean with 95% CI) of bAHI at PSG-AHI cutoffs of 5, 15, and 30 (all subjects, N = 78).

Cutoff	Accuracy	Sensitivity	Specificity	PPV	NPV	LR+	LR-	Kappa	AUROC
**5**	0.564 (0.447, 0.676)	1.000 (0.918, 1.000)	0.029 (0.001, 0.149)	0.558 (0.441, 0.672)	1.000 (0.025, 1.000)	1.029 (0.973, 1.090)	0.000 (0.000, 0.000)	0.031 (0.000, 0.092)	0.929 (0.876, 0.980)
**15**	0.808 (0.703, 0.888)	0.931 (0.772, 0.992)	0.735 (0.589, 0.850)	0.675 (0.509, 0.814)	0.947 (0.822, 0.994)	3.509 (2.179, 5.651)	0.094 (0.024, 0.361)	0.618 (0.451, 0.785)	0.955 (0.903, 1.000)
**30**	0.910 (0.824, 0.963)	0.714 (0.419, 0.916)	0.953 (0.869, 0.990)	0.769 (0.462, 0.950)	0.939 (0.849, 0.983)	15.238 (4.809, 48.286)	0.300 (0.131, 0.688)	0.687 (0.470, 0.903)	0.961 (0.919, 1.000)

bAHI = Belun apnea-hypopnea index; PSG-AHI = polysomnography apnea-hypopnea index; PPV = positive predictive value; NPV = negative predictive value; LR+ = positive likelihood ratio; LR- = negative likelihood ratio; Kappa = Cohen’s Kappa coefficient; AUROC = area under receiver operator characteristic curve.

**Table 3:** Contingency table of OSA severity measured by PSG and BSP (all subjects, N = 78).

		PSG			
		No OSA 35 (45%)	Mild 14 (18%)	Moderate 15 (19%)	Severe 14 (18%)
**BSP**	**No OSA 1 (1%)**	1 (1%)	0 (0%)	0 (0%)	0 (0%)
	**Mild 37 (47%)**	28 (36%)	7 (9%)	2 (2%)	0 (0%)
	**Moderate 27 (35%)**	6 (8%)	7 (9%)	10 (13%)	4 (5%)
	**Severe 13 (17%)**	0 (0%)	0 (0%)	3 (4%)	10 (13%)

BSP = Belun Sleep Platform; PSG = polysomnography; No OSA = AHI < 5; Mild OSA = AHI 5 to < 15; Moderate OSA = AHI 15 to < 30; Severe OSA = AHI ≥ 30

**Table 4:** Performance (mean with 95% CI) of bAHI at PSG-AHI cutoff of 15 for subjects taking heart-rate affecting medicines and subjects with comorbidities.

Effect	Accuracy	Sensitivity	Specificity	PPV	NPV	LR+	LR-	Kappa
**Medications (N = 25)**	0.840 (0.639, 0.955)	0.846 (0.546, 0.981)	0.833 (0.516, 0.979)	0.846 (0.546, 0.981)	0.833 (0.516, 0.979)	5.077 (1.403, 18.374)	0.185 (0.050, 0.677)	0.679 (0.392, 0.967)
**Comorbidities (N = 64)**	0.828 (0.713, 0.911)	0.923 (0.749, 0.991)	0.763 (0.598, 0.886)	0.727 (0.545, 0.867)	0.936 (0.786, 0.992)	3.897 (2.179, 6.971)	0.101 (0.026, 0.386)	0.658 (0.479, 0.838)

bAHI = Belun apnea-hypopnea index; PSG = polysomnography; PPV = positive predictive value; NPV = negative predictive value; LR+ = positive likelihood ratio; LR- = negative likelihood ratio; Kappa = Cohen’s Kappa coefficient

### Combining STOP-Bang and bAHI

**Fig 6:**
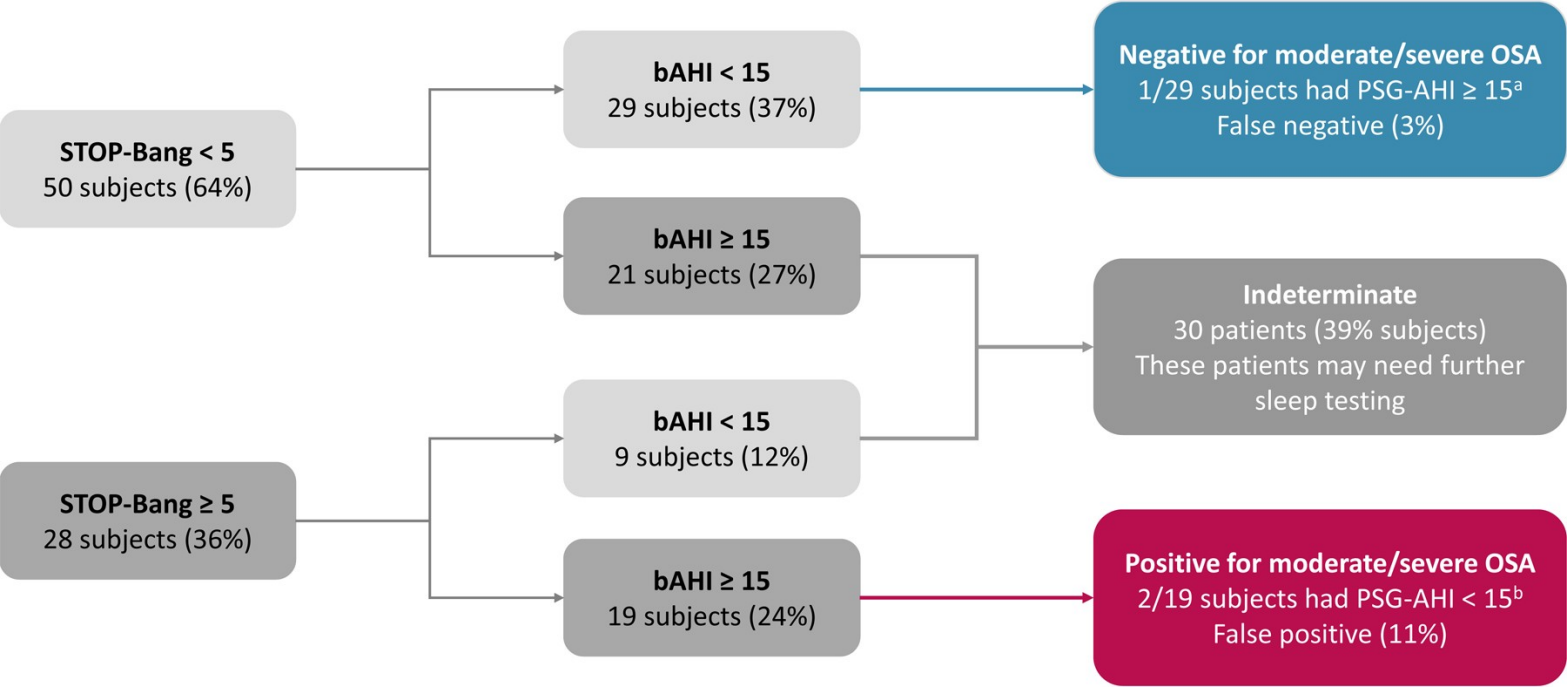
The combined diagnostic algorithm using STOP-Bang cutoff of 5 and bAHI cutoff of 15 events/h curtailed the need for further sleep testing by 61%. bAHI = Belun apnea-hypopnea index. ^a^ This false negative case had a PSG-AHI of 17.6 events/h; ^b^ These two false positive cases had mild OSA with PSG-AHI ≥ 5 events/h.

We assessed the performance of a diagnostic algorithm incorporating STOP-Bang with a bAHI cutoff of 15 events/h. We tested STOP-Bang score cutoffs of 3, 4, and 5, and a cutoff of 5 had the best performance in stratifying subjects into three categories: (1) positive for moderate or severe OSA (STOP-Bang ≥ 5 with bAHI ≥ 15 events/h); (2) negative for moderate or severe OSA (STOP-Bang < 5 with bAHI < 15 events/h); and (3) indeterminate (STOP-Bang < 5 with bAHI ≥ 15 events/h, or STOP-Bang ≥ 5 with bAHI < 15 events/h). With this combined diagnostic algorithm, 61% of the subjects need no further sleep testing (Fig 6). Compared to the STOP-Bang cutoff of 5 alone (Table 5), this diagnostic algorithm using STOP-Bang cutoff of 5 and bAHI cutoff of 15 events/h yielded better performance in assessing moderate to severe OSA (original dataset, Table 6). Internal validation of the diagnostic algorithm was performed by bootstrapping with 1,000 samples which showed small 95% bootstrapped confidence intervals around the original coefficients (bootstrapping re-sampling, Table 6).

**Table 5:** Performance (mean with 95% CI) of STOP-Bang score of 5 at PSG-AHI cutoffs of 5, 15, and 30 (N = 78).

Cutoff	Accuracy	Sensitivity	Specificity	PPV	NPV	LR+	LR-	Kappa
**5**	0.705 (0.591, 0.803)	0.558 (0.399, 0.709)	0.886 (0.733, 0.968)	0.857 (0.673, 0.960)	0.620 (0.472, 0.753)	4.884 (1.870, 12.753)	0.449 (0.349, 0.712)	0.427 (0.244, 0.610)
**15**	0.731 (0.618, 0.825)	0.621 (0.423, 0.793)	0.796 (0.657, 0.898)	0.643 (0.441, 0.814)	0.780 (0.640, 0.885)	3.041 (1.633, 5.664)	0.477 (0.293, 0.775)	0.420 (0.211, 0.628)
**30**	0.641 (0.524, 0.747)	0.500 (0.230, 0.770)	0.672 (0.543, 0.784)	0.250 (0.107, 0.449)	0.860 (0.733, 0.942)	1.524 (0.811, 2.862)	0.744 (0.429, 1.291)	0.124 (0.000, 0.332)

bAHI = Belun apnea-hypopnea index; PSG = polysomnography; PPV = positive predictive value; NPV = negative predictive value; LR+ = positive likelihood ratio;

LR- = negative likelihood ratio; Kappa = Cohen’s Kappa coefficient.

**Table 6:** Performance (mean with 95% CI) of the combined approach using STOP-Bang cutoff of 5 and bAHI cutoff of 15 (N = 48).

Methods	Accuracy	Sensitivity	Specificity	PPV	NPV	LR+	LR-	Kappa	AUROC
**Original dataset**	0.938 (0.828, 0.987)	0.944 (0.727, 0.999)	0.933 (0.779, 0.992)	0.895 (0.669, 0.987)	0.966 (0.822, 0.999)	14.170 (3.700, 54.300)	0.060 (0.009, 0.401)	0.868 (0.724, 1.000)	0.939 (0.868, 1.000)
**1,000 Bootstrapping re-sampling**	0.938 (0.860, 1.000)	0.944 (0.813, 1.000)	0.934 (0.839, 1.000)	0.894 (0.722, 1.000)	0.965 (0.886, 1.000)	14.662* (5.600, ∞)	0.060 (0.000, 0.200)	0.866 (0.698, 1.000)	0.978 (0.935, 1.000)

* Median of LR+ was provided as the use of mean produced infinite LR+.

## Discussion

This study assessed the performance of BSP in detecting OSA in adult patients referred to sleep labs for in-lab PSG. Unlike the proof-of-concept study, this cohort included patients taking medications known to affect heart rate and those with non-arrhythmic comorbidities. The bAHI and bTST derived from BSP correlated well with PSG-AHI and PSG-TST with a correlation coefficient of 0.888 and 0.967, respectively. The use of heart rate-affecting medications did not negatively influence the performance of BSP testing. Despite the presence of a relatively low overall OSA prevalence and an unusually high female-male ratio uncharacteristic for an in-lab study population, the performance of BSP remains reasonably robust.

This study again demonstrated that although BSP has a good overall performance, diagnostic discordance exists. BSP tends to overestimate AHI in individuals with AHI under 15 events/h and underestimate AHI in those with an AHI over 15 events/h [32]. Similar diagnostic discordances between PSG-AHI and HSAT respiratory event index have been described in other PAT/PPG-based devices including WatchPAT and NightOwl [16, 32, 42–44]. This overestimation of AHI in the low AHI range is likely due to the identification of RERAs and autonomic arousals from other causes by the BSP algorithm. On the other hand, the underestimation at high AHI range may be due to misses of the respiratory events when consecutive respiratory events occur over a short period, or this may be attributable to the rejection of motion artifact-related poor PPG signals in patients with very severe OSA. Overall, BSP demonstrated satisfactory performance in detecting moderate or severe OSA despite the presence of severity discordance, which is more prominent in patients with no OSA or mild OSA.

### Compared to other PAT/PPG-based devices

Several studies have investigated the performance of PAT/PPG-based HSAT. Choi et al. used WatchPAT-100 in 25 subjects and showed a sensitivity and specificity of 0.810 and 0.770 in a population with a prevalence of 68% for AHI ≥ 15 events/h [45]. Li et al. studied WatchPAT-200 performance in 43 individuals with suspected OSA and revealed a sensitivity and specificity of 0.938 and 0.667 in a population with a prevalence of 57% for AHI ≥ 15 events/h [42]. Ioachimescu et al. recently conducted a study using WatchPAT-200 in 500 consecutive veterans and reported a sensitivity and specificity of 0.910 and 0.610 with a prevalence of 58% for AHI ≥ 15 events/h [17]. Massie et al. compared NightOwl with simultaneous PSG with automated scoring in 101 subjects and showed a sensitivity and specificity of 0.970 and 0.830 in a population with a prevalence of 59% for AHI ≥ 15 events/h [44]. The current study demonstrated a comparable sensitivity of 0.931 [95% CI, 0.772–0.992] and specificity of 0.735 [95% CI, 0.589–0.850] in a population with a relatively low prevalence of moderate to severe disease (37%).

### Methods to improve diagnostic precision in PAT/PPG-based testing

Overall, PAT/PPG-based testing has a sensitivity and specificity in the range of 0.810 to 0.970 and 0.610 to 0.830 for moderate to severe OSA and has the potential to significantly misclassify OSA severity [17, 42, 44, 45]. A couple of approaches have recently been developed to improve the performance and diagnostic concordance of PAT/PPG-based testing. Zhang et al. developed and validated a manual algorithm for visual editing of WatchPAT automated scoring and assessed its accuracy in an unselected clinical population [46]. The authors concluded that a 10–15 minute manual editing of automatic scored data can improve correlation and agreement with PSG as well as a concordance for categorical agreement of OSA severity. Alternatively, Ioachimescu et al. explored statistical models to predict AHI by using robust functional parameters from WatchPAT-200 in concert with available demographic and anthropometric data including age, gender, neck circumference, and body mass index [47]. In this cohort of 500 patients with a high pretest probability of OSA, the mean diagnostic accuracy of WatchPAT was improved to 67%, 81%, and 85% in mild, moderate-severe, or no OSA, respectively. The authors concluded that these models can be used to improve the diagnostic precision of the PAT-based testing, thus ameliorating the high rates of misclassification for OSA presence or disease severity.

Our study demonstrated that another feasible approach to improve diagnostic precision is to simply combine PAT/PPG-based testing with STOP-Bang questionnaire, which is ubiquitously used for OSA screening in clinical practice. The concept of combining a prediction score and portable testing for diagnosis and screening of OSA is not new [48–50]. Morales et al. addressed the diagnosis of severe OSA in a sleepy elderly population with a two-stage strategy using Multivariable Apnea Prediction score followed by nasal pressure-based HSAT [48]. Gurubhagavatula et al., using the same approach, screened internal medicine outpatients with hypertension for severe OSA. Both studies concluded that a two-stage approach performed better in identifying severe OSA than the single-stage approach [49]. More recently, Mashaqi et al. reported that the combined use of STOP-Bang and nocturnal oximetry measures improved the accuracy of severe OSA screening in both inpatient and outpatient settings [50]. Our combined approach provides an easy method to better predict moderate to severe OSA with high precision in the majority of subjects even in the setting of a relatively low prevalence and allows further stratification, with which an appropriate clinical course of action can be consequently taken. This proposed approach can potentially curtail the need for more sophisticated sleep testing, which may lead to significant healthcare cost savings.

### Strengths and weaknesses

This study is the first investigation to use a combination of a sleep questionnaire and a medical-grade wearable using neural network algorithm to identify OSA. One of the strengths of this study is the concurrent recording of both in-lab PSG and BSP. All sleep studies were manually scored based on the most current AASM scoring manual by one of our two senior registered sleep technicians and reviewed by an experienced board-certified sleep specialist. The scoring technicians and the sleep specialist reviewing the sleep studies were blinded to the BSP results.

As far as the limitations are concerned, readers should take into account that this sampled population, unlike a typical sleep lab population with high OSA prevalence, skewed towards females and those with no OSA or mild OSA. This could have resulted from a high percentage of male patients with severe OSA declining to consent. This made the comparison of this study with other studies difficult, but nonetheless allows us an opportunity to project how BSP may perform in a non-sleep clinic where the prevalence of moderate to severe OSA is relatively low. Another limitation of this study is that this study was not performed in an unattended home setting but a sleep lab setting. Further investigation in the home setting in comparison to established HST devices is warranted. Finally, this diagnostic algorithm combining STOP-Bang and bAHI requires further validation in various patient populations and clinical settings.

## Conclusions

BSP is a promising wearable sleep technology that can potentially be incorporated into clinical practice for detecting OSA. Combining STOP-Bang cutoff of 5 with a bAHI cutoff of 15 events/h improves prediction precision and diagnostic concordance in detecting moderate or severe OSA, which may be used to streamline the clinical practice. Further clinical investigation for the diagnosis and screening of moderate to severe OSA using BSP with or without the combination with STOP-Bang is warranted.

## Supporting information

S1 Data (XLS)
