# Correction: Detection of obstructive sleep apnea using Belun Sleep Platform wearable with neural network-based algorithm and its combined use with STOP-Bang questionnaire

**DOI:** 10.1371/journal.pone.0303713

**Published:** 2024-05-09

**Authors:** Eric Yeh, Eileen Wong, Chih-Wei Tsai, Wenbo Gu, Pai-Lien Chen, Lydia Leung, I-Chen Wu, Kingman P. Strohl, Rodney J. Folz, Wail Yar, Ambrose A. Chiang

In Table 1, the numbers in the rows White and Asian are swapped. Please see the correct Table 1 here.

**Table 1:** Summary of Subject Characteristics and PSG Results.

Parameter		OSA Severity					*P*-value
	ALL	No OSA	Mild OSA	Moderate OSA	Severe OSA	All OSA	
**Subject (%)**	78 (100%)	35 (45%)	14 (18%)	15 (19%)	14 (18%)	43 (55%)	-
**Gender (%)** **Male** **Female**	27 (35%) 51 (65%)	9 (12%) 26 (33%)	3 (4%) 11 (14%)	10 (13%) 5 (6%)	5 (6%) 9 (12%)	18 (23%) 25 (32%)	< 0.05^b^
**Age (y)**	51.5 ± 13.4	49 ± 14	47 ± 12	54 ± 11	54 ± 15	52 ± 13	0.24^a^
**Race (%)** **Black** **White** **Asian** **Others**	41 (53%) 30 (38%) 4 (5%) 3 (4%)	17 (22%) 13 (17%) 3 (4%) 2 (2%)	10 (13%) 3 (4%) 0 (0%) 1 (1%)	6 (8%) 8 (10%) 1 (1%) 0 (0%)	8 (10%) 6 (8%) 0 (0%) 0 (0%)	24 (31%) 17 (22%) 1 (1%) 1 (1%)	0.57^b^
**BMI (kg/m** ^**2**^ **)**	36.1 ± 10.1	31.4 ± 7.9	42.5 ± 10.2	39.1 ± 10.6	38.1 ± 9.8	39.9 ± 10.1	<0.01^a^
**STOP-Bang**	3.9 ± 1.8	3.1 ± 1.6	3.8 ± 1.5	5.3 ± 1.5	4.4 ± 1.9	4.5 ± 1.7	<0.01^a^
**AHI (events/h)**	14.5 ± 17.0	2.4 ± 1.5	8.3 ± 2.0	21.1 ± 3.9	43.6 ± 16.9	24.9 ± 20.0	<0.001^a^
**CAI (events/h)**	1.39 ± 3.5	0.40 ± 0.7	0.77 ± 1.8	2.11 ± 3.9	3.71 ± 6.6	2.19 ± 4.6	<0.05^a^

AHI = apnea-hypopnea index; BMI = body mass index; CAI = central apnea index; OSA = obstructive sleep apnea; PSG = polysomnography. No OSA = AHI < 5; Mild OSA = AHI 5 to < 15; Moderate OSA = AHI 15 to < 30; Severe OSA = AHI ≥ 30.

Mean ± standard deviation for age, BMI, AHI, and STOP-Bang.

Numbers of subjects (%) for gender and race.

a Kruskal-Wallis test to compare mean ranks in age, BMI, STOP-Bang, AHI, and CAI among No OSA, Mild OSA, Moderate OSA, and Severe OSA groups.

b Mantel-Haenszel Chi-Squared test to compare frequencies in gender, and race among No OSA, Mild OSA, Moderate OSA, and Severe OSA groups.
